# Quantitative Damage Detection and Sparse Sensor Array Optimization of Carbon Fiber Reinforced Resin Composite Laminates for Wind Turbine Blade Structural Health Monitoring

**DOI:** 10.3390/s140407312

**Published:** 2014-04-23

**Authors:** Xiang Li, Zhibo Yang, Xuefeng Chen

**Affiliations:** 1 State Key Laboratory for Manufacturing Systems Engineering, Xi'an Jiaotong University, Xi'an 710049, China; E-Mails: lixiangworks@gmail.com (X.L.); phdapple@mail.xjtu.edu.cn (Z.Y.); 2 School of Mechanical Engineering, Xi'an Jiaotong University, Xi'an 710049, China

**Keywords:** structural health monitoring, sensor array optimization, composite laminates, second generation wavelet, Lamb wave, wind turbine blade

## Abstract

The active structural health monitoring (SHM) approach for the complex composite laminate structures of wind turbine blades (WTBs), addresses the important and complicated problem of signal noise. After illustrating the wind energy industry's development perspectives and its crucial requirement for SHM, an improved redundant second generation wavelet transform (IRSGWT) pre-processing algorithm based on neighboring coefficients is introduced for feeble signal denoising. The method can avoid the drawbacks of conventional wavelet methods that lose information in transforms and the shortcomings of redundant second generation wavelet (RSGWT) denoising that can lead to error propagation. For large scale WTB composites, how to minimize the number of sensors while ensuring accuracy is also a key issue. A sparse sensor array optimization of composites for WTB applications is proposed that can reduce the number of transducers that must be used. Compared to a full sixteen transducer array, the optimized eight transducer configuration displays better accuracy in identifying the correct position of simulated damage (mass of load) on composite laminates with anisotropic characteristics than a non-optimized array. It can help to guarantee more flexible and qualified monitoring of the areas that more frequently suffer damage. The proposed methods are verified experimentally on specimens of carbon fiber reinforced resin composite laminates.

## 1. Introduction

Wind energy is regarded as a key resource for meeting planned carbon emission reduction targets and achieving energy supply source diversification [1,2]. The growing interest in wind energy has led to the rapid expansion of wind farms [2,3]. The growth of wind power has increased interest in the operational safety and maintenance of wind turbines. As wind turbines are often located at remote locations that may be difficult to access, and as the size of the wind turbine structures used has increased and their initial investment costs have increased correspondingly, there has been a gradual increasing need for structural health monitoring (SHM) [4]. Condition monitoring and fault diagnosis of wind turbine structures are of high priority. Wind turbine structures can be damaged by moisture absorption, fatigue, operational failure, wind gusts or lightning strikes [5].

As the basic material of the structure, composites have played important roles in the wind power plant area, especially for wind turbine blades. Along with their expanding applications, presence in essential parts and a higher proportion in structures, composites have also become indicators for appraising advancements in structure design. This makes SHM indispensable for ensuring safety, implementing timely maintenance, avoiding disastrous events and lowering costs [6–10].

Numerous approaches have been utilized in recent years to perform SHM on different structures, including composites [11]. They can be broadly classified into two categories: passive methods and active methods. Passive SHM methods (such as acoustic emission, impact detection, strain measurement, *etc.*) have been studied longer and are relatively mature, however, they suffer from several drawbacks (need for continuous monitoring, indirect inference of damage existence, *etc.*) which limit their utility. Active SHM methods are currently of greater interest due to their ability to perform on-demand interrogation of a structure while the structure is still in service. One promising active SHM method utilizes arrays of piezoelectric lead zirconate titanate (PZT) sensors bonded to a structure for both transmitting and receiving ultrasonic waves in order to achieve damage detection [12,13]. When used to interrogate thin-plate structures, the PZT are effective guided wave transducers which couple their in-plane motion with the guided wave particle motion on the material surface.

To date several research projects to realize more effective detection of the health status of structures have been done. Su, Ye and Lu provided a comprehensive review of the Lamb wave-based damage identification approaches for composite structures [14]. Giurgiutiu presented a number of experimental results for damage detection in simple flat unidirectional and quasi-isotropic composite specimens [15]. Tang, Winkelmann, *et al.* correlated the contour area changes with the so-called characteristic damage state in composite laminates under tensile fatigue [16].

In current SHM researches, the methods based on PZTs and active Lamb waves are the most frequently and broadly adopted effective methods for crucial structures due to their sensitivity for small sized damages. Lamb waves can be propagated for a long distance without significant amplitude attenuation in plate structures, which offers good performance for monitoring large area structures like wind turbine blades. Unfortunately, the Lamb wave is unavoidably polluted from multiple interference sources and strong noise in the anisotropic composite laminate structure, which needs more precise and efficient advanced signal processing and feature extraction methods to accurately identify damage information, which in turn offers more reasonable feature parameters [17,18].

Quite a few studies have addressed the effect of load on the properties of Lamb waves. In composites, the signals under consideration are known to be non-stationary. Wavelet analysis, as a widely validated method for processing non-stationary signals, has been adopted by many researchers to date, e.g., to mention a few, Liu *et al.* [19], Wang *et al.* [20], Ramadas *et al.* (2011) [21], Park, Gyuhae, *et al.* [22], Huang *et al.* [23], Yan *et al.* [24], Li *et al.* [25], Second generation wavelets are also applied in the signal analysis of machine faults [26] and composite damage identification [27]. However, to the best of our knowledge, the tomography technique and interpretation to visually and quantitatively identify the location of the damage shown in this article are both relatively novel concepts.

A technique based on Improved Redundant Second Generation Wavelet Transform (IRSGWT) is presented in this study. The Second Generation Wavelet Transform adopting lifting scheme was proposed by Sweldens as a new method of wavelet construction [28]. The application of the lifting scheme for rotating machinery fault diagnosis has been reported by Duan *et al.*, Li *et al.* and Chen *et al.* [29–31].

In the complex composite laminate structures of wind turbine blades, active monitoring faces a more serious and more complicated noise problem, so an appropriate denoising method must be used. For large scale structures such as wind turbine blades, how to minimize the amount of sensors while maximizing accuracy is also a key issue. Therefore, on the basis of the former study [10], the present paper presents a practical and effective damage quantitative monitoring technique for Carbon Fiber Reinforced Resin Composites Laminates (CFRRCL) as an important area of wind turbine blade structure research. The second section illustrates a view of the general damage analysis of wind turbine blades with composite structures. The third section proposes the theory of the IRSGWT and the principle of IRSGWT denoising based on the neighboring coefficients. The fourth section presents and discusses the sparse sensor array optimization of composite laminates for wind turbine blade applications. The fifth section shows the experimental set-up and the analysis of the results. The feasibility of the entire system is then validated by applying it to locating damage in an anisotropic composite laminate. The last section summarizes in the conclusions the key findings of this paper and discusses future work.

## 2. General Damage Analysis of Wind Turbine Blades with Composite Laminate Structures

### 2.1. The Requirement of SHM for Wind Turbine Structures

**Figure 1.**
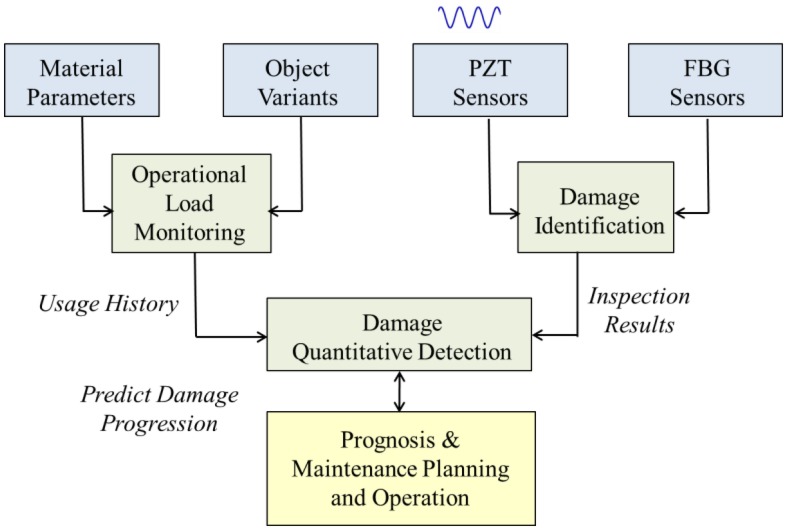
System architecture of SHM [34].

Wind energy has attracted great attention because of the recent energy crisis and the pressing need for clean energy sources. The worldwide wind capacity reached 296,255 MW by the end of June 2013, out of which 13,980 MW were added just in the first six months of 2013. With all this tremendous installed capacity, development and investment, major issues of wind power system are the concerns about operational safety, fault diagnosis and maintenance [32]. Wind turbines are hard-to-access structures, and they are usually located in remote places. These characteristics and working conditions, especially for composite materials and rotating blades, make the SHM for wind power systems more difficult and complex. With a reliable SHM system, a promising monitoring, maintenance and repair strategy for wind turbines can be planned [33]. A typical SHM system architecture is shown in Figure 1.

### 2.2. The Damage Analysis of Wind Turbine Structures

**Figure 2.**
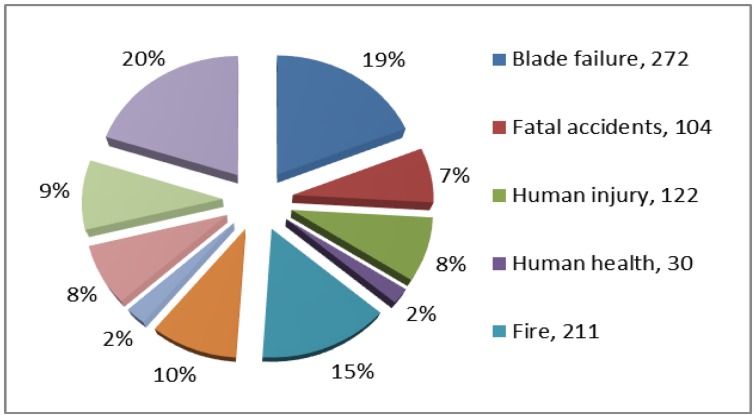
Distribution of wind power generator damage types.

A SHM system that is reliable, low cost and integrated into the wind turbine structure may reduce wind turbine life-cycle costs and make wind energy more affordable. Figure 2 covers every documented case of wind turbine related accidents and incidents that could be found and confirmed via press reports or official information releases up to 30 September 2013.

It shows that 19% of all wind turbine related incidents are on account of “blade failure”. While other incidents are mentioned, such as fires (15%), structural failures (10%), environmental damage (9%), human injury (8%), fatal accidents (7%), transport (8%), ice throw incidents (2%), and human health (2%) the statistical analysis distinctly shows that blade failure is the most frequent wind turbine accident [35,36]. This is in line with a recent survey by GCube [37], the largest provider of insurance for renewable energy schemes in the USA.

Therefore, the wind turbine blade plays crucial role in both the safety and operational confidence of a wind turbine system. Several types of damage in the load-carrying structural blades, such as the skins and the main spar, are included in the processes involved in the failure of specific blades. The types of damage studied in the current study were categorised as follows:

Type 1: Damage formation and growth in the adhesive layer joining skin and main spar flanges (skin/adhesive debonding and/or main spar/adhesive layer debonding);
Type 2: Damage formation and growth in the adhesive layer joining the up- and downwind skins along leading and/or trailing edges (adhesive joint failure between skins);
Type 3: Damage formation and growth at the interface between face and core in sandwich panels in skins and main spar web (sandwich panel face/core debonding);
Type 4: Internal damage formation and growth in laminates in skin and/or main spar flanges, under a tensile or compression load (delamination driven by a tensional or a buckling load);
Type 5: Splitting and fracture of separate fibres in laminates of the skin and main spar (fibre failure in tension; laminate failure in compression);
Type 6: Buckling of the skin due to damage formation and growth in the bond between skin and main spar under compressive load (skin/adhesive debonding induced by buckling, a specific type 1 case);
Type 7: Formation and growth of cracks in the gel-coat; debonding of the gel-coat from the skin (gel-coat cracking and gel-coat/skin debonding).
Type 8: Damage focused by lightning strikes.

**Figure 3.**
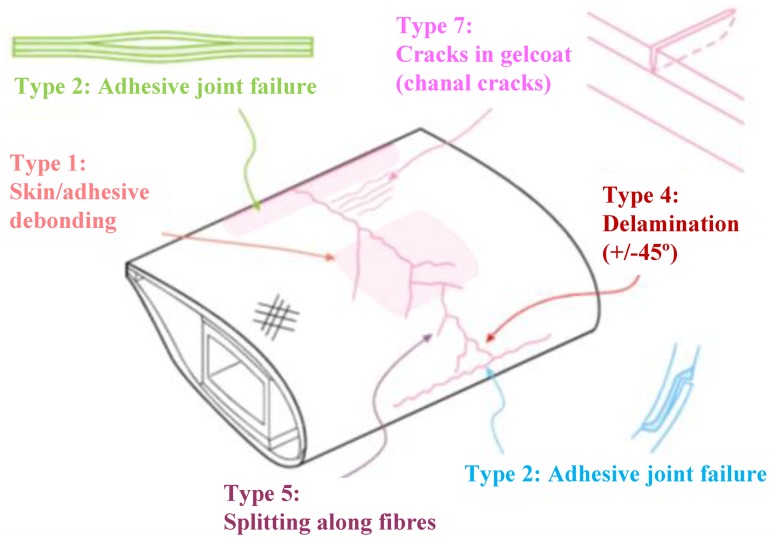
A view of some of the damage types found during the examination of the downwind skin of test section subjected to a compressive load [38]. Damages to the adhesive layers: Types 1 (skin/adhesive debonding) and 2 (adhesive joint failure between skins) at the leading as well as the trailing edge. Damage to the downwind skin under compressive load: Types 4 (delamination driven by a buckling load), 5 (laminate failure in compression) and 7 (gel-coat cracking and gel-coat/skin debonding).

A sketch of some of the damage types found during examinations at wind farms is shown in Figure 3 [38].

### 2.3. The Frequently Seen Locations of Damage on Wind Turbine Blades

**Figure 4.**
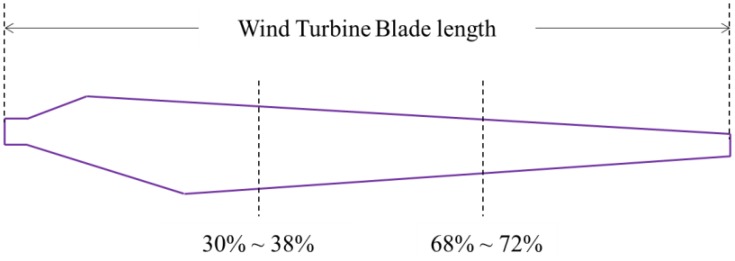
The spanwise location of a wind turbine blade that is likely to damage [39].

According to the statistical results from the damaged blades in wind-farm, most blade damages are quite regular, and the fractured locations are near the blade root or a place located at 1/3 of the blade length from the tip to the root. The remaining damage situations are relatively less frequent. In the long term monitoring of wind turbines, the causes of the fracture are also typically the same. Figure 4 illustrates the damage-prone locations on the blade, while Ref. [39] shows an example of damage.

## 3. IRSGWT-Based Denoising Approach for Composite Laminate Structures

Since composite laminates are complex in lay-up, which evidently means they have layering and anisotropy characteristics, there are seldom reliable and mature damage monitoring methods, especially measures for detecting large areas and irregular structures. In current SHM studies, the most frequently applied effective method is still based on PZTs and active Lamb waves thanks to its sensitivity for small size damages, such as cracks or delaminations. In composite laminates, the Lamb wave is unavoidably polluted by multiple interference sources and strong noise, which requires much more precise and efficient advanced signal processing and feature extraction methods to accurately identify damage information which in turn supplies more reasonable feature parameters. Unfortunately, the dispersion and complicated transition phenomena in composite laminate structures are complex to analyze and interpret. Material anisotropy intensifies this difficulty [18].

**Figure 5.**
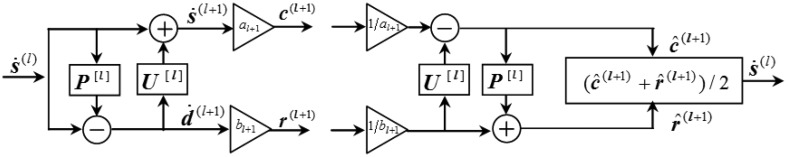
The forward and inverse transform of IRSGWT.

In the Introduction section, we have mentioned the high potential of the second generation wavelet transform (SGWT) and redundant second generation wavelet transform (RSGWT) in both rotor machine and composite structure applications [40]. In this section, we provide a review of the Improved RSGWT (IRSGWT) algorithm, which increases the capability for the signal denoising on the basis of the RSGWT we have studied previously [10]. Furthermore, aiming at extracting the weak signals from the polluted raw signals propagating in composite laminates for wind turbine blade applications, a proper IRSGWT algorithm based on neighboring coefficients is presented to help overcome the shortcomings of conventional wavelet threshold denoising [40–42]. Through adding the normalization factors, the IRSGWT is proposed to control the error propagation caused by RSGWT. The forward and inverse transforms of IRSGWT are shown in Figure 5.

The *a_l_*_+1_ and *b_l_*_+1_ are the normalization factors at level *l* + 1. The decomposition results of the approximation signal *ṡ*^(^*^l^*^)^ at level *l* with IRSGWT are expressed as follows:

(1) $${\dot{d}}^{\left(l+1\right)}={\dot{s}}^{\left(l\right)}-P^{\left(l\right)}{\dot{s}}^{\left(l\right)}$$

(2) $${\dot{s}}^{\left(l+1\right)}={\dot{s}}^{\left(l\right)}-U^{\left(l\right)}{\dot{d}}^{\left(l+1\right)}$$

(3) $$r^{\left(l+1\right)}=b_{l+1}{\dot{d}}^{\left(l+1\right)}$$

(4) $$c^{\left(l+1\right)}=a_{l+1}{\dot{s}}^{\left(l+1\right)}$$

where *ḋ*^(^*^l^*^+1)^ and *ṡ*^(^*^l^*^+1)^ are computed by the classical redundant lifting scheme. *r*^(^*^l^*^+1)^ and *c*^(^*^l^*^+1)^ are the detail signal and approximation signal at level *l* + 1 using IRSGWT.

Suppose the total energy of the approximation signal *ṡ*^(^*^l^*^)^ is described as:

(5) $$E_{s_{l}}=\sum_{i=1}^{n}{\left[{\dot{s}}_{i}^{\left(l\right)}\right]}^{2}$$

The total energies of *ṡ*^(^*^l^*^+1)^ and *ṡ*^(^*^l^*^+1)^ are given by *E*_*s*_*l*+1__ and *E*_*d*_*l*+1__, respectively:

(6) $$E_{S_{l+1}}=\sum_{i=1}^{n}{\left[{\dot{s}}_{i}^{\left(l+1\right)}\right]}^{2}$$

(7) $$E_{d_{l+1}}=\sum_{i=1}^{n}{\left[{\dot{d}}_{i}^{\left(l+1\right)}\right]}^{2}$$

The total energies of *c*^(^*^l^*^+1)^ and *r*^(^*^l^*^+1)^ are expressed by *E*_*c*_*l*+1__ and *E*_*r*_*l*+1__, respectively:

(8) $$E_{c_{l+1}}=\sum_{i=1}^{n}{\left[{\dot{c}}_{i}^{\left(l+1\right)}\right]}^{2}$$

(9) $$E_{r_{l+1}}=\sum_{i=1}^{n}{\left[{\dot{r}}_{i}^{\left(l+1\right)}\right]}^{2}$$

When the error propagation is controlled, the total energy of original signal should be approximately equal to that of decomposition results, *i.e.*, *E_s_l__* = *E*_*c*_*l*+1__ + *E*_*r*_*l*+1__. Furthermore, the approximation signal *ṡ*^(*l*+1)^ cannot give the error propagation in decomposition results using a redundant lifting scheme. The normalization factors are calculated by the following equations:

(10) $$a_{l+1}=1$$

(11) $$b_{l+1}=\sqrt{({E_{s}}_{l}-{E_{s}}_{l+1})/{E_{d}}_{l+1}}$$

## 4. Sensor Array Sparse Optimization of Composite Laminate Structures

The performance of in-service composite laminate structures is influenced by degradation resulting from exposure to severe environmental conditions or damage resulting from external events, such as impact, fatigue, operator abuse or neglect. It is desirable to detect the signs of damage as early as possible and thus allow proper maintenance since the failure of an important large structure may cause a massive disaster [43–46]. This section presents the sparse sensor requirement and scheme of composite laminates for wind turbine blade SHM applications. If the SHM system for a large structure has a large amount of sensors and is run in real-time operations, the system may possibly suffer computational overburden. One of the challenges is how to design, optimize and manage the sensor array distribution. For large scale structures such as the composite laminates of wind turbine blades, how to minimize the amount of sensors while maintaining accuracy is a crucial issue [47,48]. According to the analysis of Section 2, the damage area and type frequently happen in a generalized manner for wind turbine blades, possibly as fractures near the blade tip or root position. Therefore, identifying the crucial monitoring area is also relatively significant for blade health monitoring.

**Figure 6.**
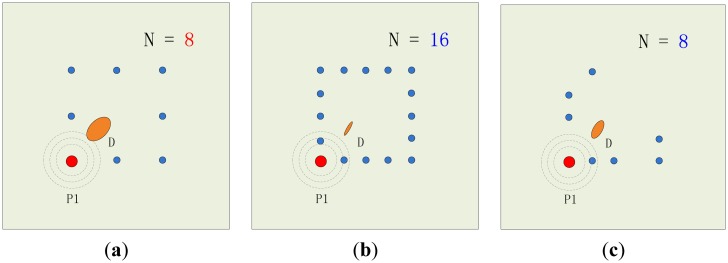
The scheme of the sparse sensor optimization method. (**a**) 8 Transducers not optimized; (**b**) Full 16 transducers; (**c**) Top 8 transducers optimized.

As an instructional work, a sparse sensor optimization scheme is proposed in this paper. In our former research, an eight transducer array configuration was adopted to monitor the occurrence of damages on composite laminates for wind turbine blades. The former array is fixed to be essentially distributed as a rectangle, which doesn't consider the efficiency of each transducer's performance contribution. The situation varies according to the location of the arrays which are placed on different sections of the work piece, as well as the location and type of damage. Moreover, when the number of the transducers increases to sixteen, the accuracy rises accordingly, but more attention must be paid to the transducers' location contributions for better performance and error control with such a heavy distribution number. When this monitoring method is applied to several areas on composite laminates, we expect to use the sparse array to monitor same size or even larger areas. The evaluation indexes of energy contribution index, accuracy affect index and average location error are given to select the best transducer spots for the monitoring area. The optimized eight transducer configuration would have the closest accuracy compared with a full sixteen transducer array. The scheme of the sparse sensor optimization method is shown as Figure 6. Figure 6a illustrates the averagely distributed eight transducer array without considering the performance contribution, Figure 6b shows the broadened 16 transducer array without considering the performance contribution, while Figure 6c indicates a vision that when the array location is optimized, the number of transducers could be decreased, the distribution is possibly no longer average, but the accuracy remains at a high level beyond the average one. The approach can greatly reduce the complexity in large scale laminate monitoring, raise the accuracy and manage the risk of error. The sensor sparse optimization algorithm procedure is as follows:

Step 1. Confirm the number of damages.
Step 2. Compute the probabilistic reconstruction algorithm tomography map *Z* by using the complete measuring transducers set.
Step 3. Compute the index value of each transducer spot and give a contribution and performance rank for each transducer spot.
Step 4. Recompute the tomography of each eight sparse spots among all sixteen and compare results.
Step 5. Repeat step 4 iteratively to pick the top eight transducers as the top contributing group with the best index performance.
Step 6. Select the optimized top group of all the compared groups and recompute the sparse probabilistic reconstruction algorithm tomography map Z′.

## 5. Experiments and Result Analysis of Composite Laminate Quantitative Damage Diagnosis

### 5.1. Experimental Setup

Lamb waves, which can propagate a relatively far distance in laminated composites with a good high attenuation ratio performance, display a great sensibility to the occurrence of damage in their travel path [49,50]. A wide area can accordingly be interrogated with a handful of transducers. Moreover, the interlaminar stresses caused by Lamb waves make detection over the complete laminate thickness possible, which offers a potential way to diagnose the internal defects as well as the surface ones. With their rapid velocity, waves reflected from boundaries may easily conceal damage-scattered components in the signals. To guarantee precision, the inspection area covered by transducers must be a relatively proper sized unit of the large structure. PZT elements give outstanding performance in Lamb wave generation and acquisition, and are appropriate for integration into a host structure as an *in-situ* actuator/sensor, thanks to their negligible mass/volume, convenient integration, excellent mechanical strength, wide frequency responses, low power consumption and acoustic impedance, as well as low cost [17]. Applications of Lamb wave generation for damage detection purpose are numerous. PZT-generated Lamb waves unavoidably contain multiple modes, which are reflected as dispersive properties. Dispersive properties of multiple wave modes throughout the thickness of the medium are not identical, even for the same mode but in different frequency ranges [51,52]. Appropriate signal processing is accordingly required. Lamb waves are already widely regarded as the most frequently used method when analyzing plate structures. A narrow frequency band sine modulated wave packet is applied as an excitation signal for actuating a Lamb wave in this paper; it is generated by:

(12) $$u(t)=A[H(t)-H(t-n/f_{\text{centre}})][1-\cos(2\pi f_{c}t/n)]\sin2\pi f_{\text{centre}}t$$

where *A* is the signal amplitude, *n* is the peak number, *f_centre_* is central frequency and *H*(t) is the Heaviside Step Function.

**Figure 7.**
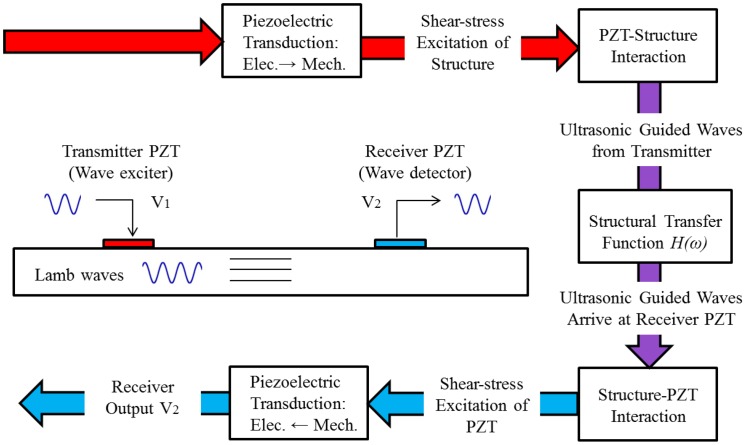
The flowchart of a PZT pitch-catch configuration for Lamb waves.

It is verified that there are two fundamental modes, A_0_ and S_0_, propagating in composite laminate structures [53]. Their phase/group velocity is dependent on the algebraic product of the laminate thickness and the mode central frequency [6]. This suggests that the central frequency for a product of frequency-thickness up to 1 MHz·mm could reasonably guarantee that no higher modes will be required for analyzing the specimen, which removes part of the analysis difficulties. In this experiment, *f_c_* = 30 kHz and *n* = 5. The power and energy transduction flow chart for a complete pitch-catch setup is shown in Figure 7.

**Figure 8.**
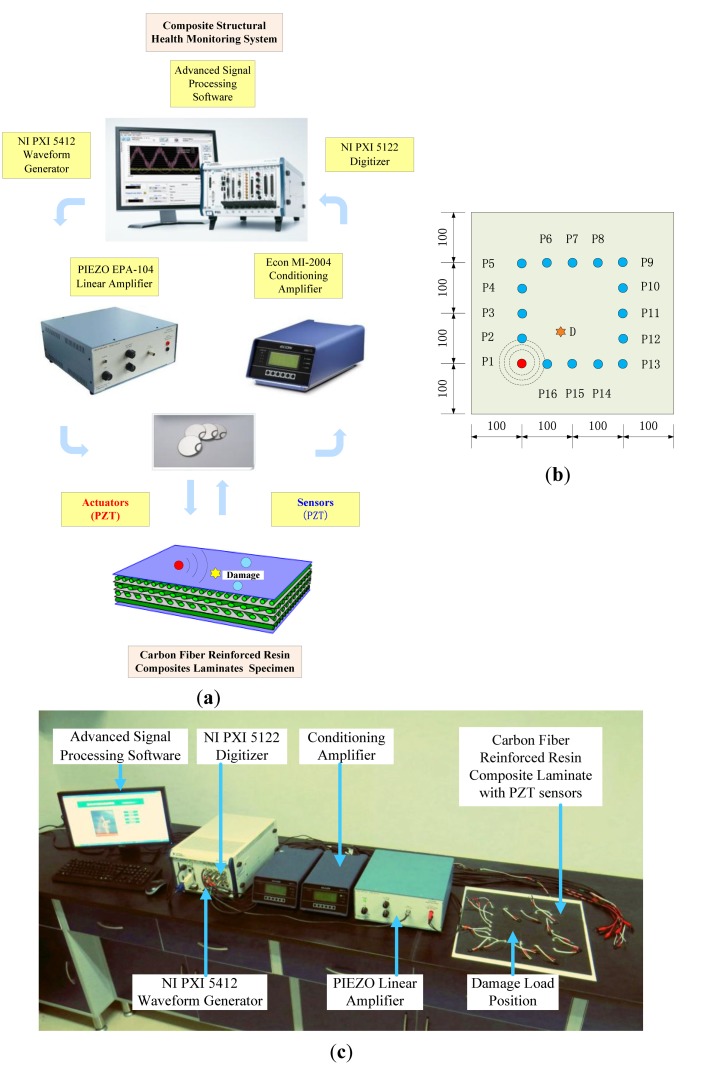
Schematic of damage detection using the proposed monitoring system with active sensor network. (**a**) Block diagram of the experimental system; (**b**) 16 PZT actuator and sensor layout on CFRRCL specimen (unit: mm); (**c**) Overall view of experiment setup.

The validation experiment is supported by composite damage monitoring system, as shown schematically in Figure 8a. It is established for emphasizing the advanced signal processing algorithm and mainly constructed on an NI PXI platform which allows further investigation of composite damage mechanisms and detection.

An experimental specimen of CFRRCL plate with dimensions 400 mm × 400 mm × 1 mm (±0.1) is employed in this research. Its lay-up contains eight plies with a configuration of [0/±0.45/90]*_s_*. The carbon fiber material density was 1.76 g/cm^3^ and the epoxy resin density was 1.23 g/cm^3^. The carbon fiber tensile strength is 3,530 MPa and the epoxy resin tensile strength is 73 MPa. The carbon fiber modulus of elasticity for tension is 230 GPa and the epoxy resin modulus of elasticity for tension is 3.3 GPa. In the form of a mass of load, the artificial damage has a circular diameter of 5 mm, and the active sensor network is configured by an array of 16 PZTs with a diameter of 8 mm and 0.5 mm in thickness, as shown in the layout coordinates (unit: mm) in Figure 8b. An overall photoview of the experimental setup is illustrated in Figure 8c.

### 5.2. Experimental Results and Their Analysis

#### 5.2.1. Experimental Signals

**Figure 9.**
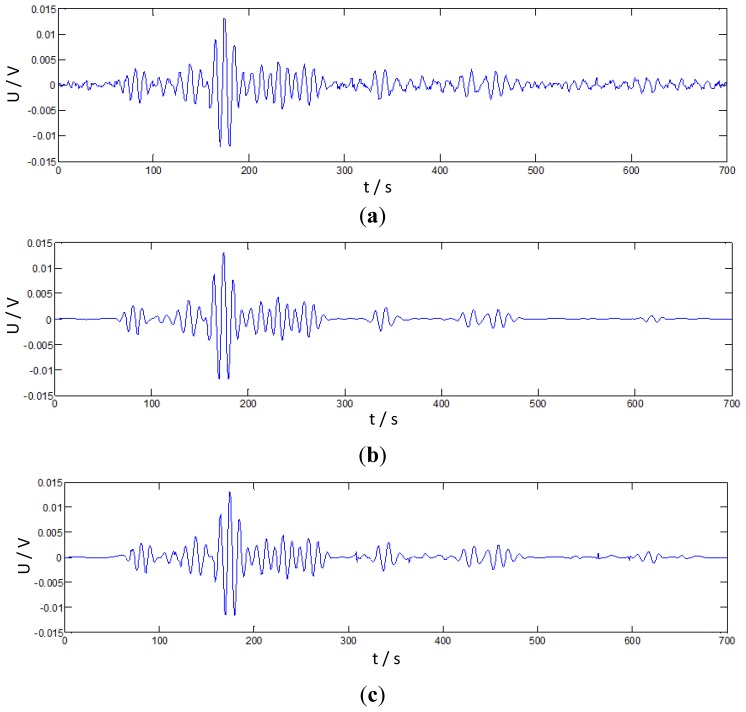
Denoising signals via actuator–sensor path P2-P10. (**a**) Raw Lamb wave damage signal via path P2-P10; (**b**) RSGWT Denoising of Lamb wave damage signal via path P2-P10; (**c**) IRSGWT Denoising of Lamb wave damage signal via path P2-P10.

In the experiment, the amplitude of the excitation signal is 10 V, the central frequency is 30 kHz and the sample rate is 300 kHz. A raw damage signal via actuator-sensor path P2-P10 as a sampling example is presented in Figure 9a. Considering the influence of the composite anisotropy property, the signal would possibly be weak. The denoising algorithm of IRSGWT based on neighboring coefficients which was discussed in Section 3 was employed in the processing procedure.

It compares with the SGWT and RSGWT denoising algorithms with neighboring coefficients and soft thresholding and hard thresholding algorithms. Because the split step is discarded in RSGWT and IRSGWT, the redundant prediction and update coefficients are calculated by padding the original prediction and update coefficients with zeros at the corresponding level. When the numbers of prediction and update coefficients vary, interval and smoothness will change, but the waveforms are similar. The numbers are properly chosen as 6 and are obtained by implementing an interpolation subdivision method. Values are [0.0117, −0.0977, 0.5859, 0.5859, −0.0977, 0.0117] and [0.0059, −0.0488, 0.2930, 0.2930, −0.0488, 0.0059], respectively [51,54]. Their denoising results are shown as Figure 9b,c. Clearly, the denoising algorithm of IRSGWT based on neighboring coefficients (NeighCoef) performs better and retains more of the original features of the raw signal.

To evaluate denoising result effects, three experienced indicators are introduced: Signal-to-Noise Ratio (SNR), Mean Square Error (MSE) and Correlation Coefficient (CC) [55,56]. The computed denoising indexes of the experimental signals also indicate the denoising algorithm of IRSGWT based on neighboring coefficients has more remarkable performance, as shown in Table 1.

As illustrated in Table 1, the IRSGWT denoising algorithm based on neighboring coefficients performs better than the others. Because the noise is possibly strong and always unknowable in the composite laminates, the best denoising solution here was adopted as our denoising approach in the experiments. In addition to this, IRSGWT has all the merits of SGWT and RSGWT, such as the construction of base wavelets can be fulfilled in the time domain, faster calculation speed, less memory use, adaptability to arbitrary signal length, the advantage of the translation invariance, which allows the same length of decomposition signals and raw signals, and it specially it removes the error propagation generated from the RSGWT. This advance can both provide more abundant information from signals and give more accurate results. The results in Table 1 also indicate that the neighboring coefficient-based denoising method was more effective than the soft threshold denoising method which was better than the hard threshold method as well.

#### 5.2.2. Damage Localization Tomography Maps

Often, actuator-sensor pair signals will be influenced by flaws, while others without flaws will not. As a result, in the defect distribution probability image, the pixel where the flaw is located will have higher probability than the others. By employing this two-dimensional probability density function, an image showing a distribution of the possible damage locations could be pictured. To combine the Lamb wave principle and tomographic imaging algorithms, a probabilistic reconstruction algorithm is introduced to provide a probabilistic-based imaging in plate-like structures, such as composite laminates [57,58]. For damage index factor, considering the anisotropy of composite laminate damage detection, and the fractal dimension of Lamb waves as a damage-sensitive feature is adopted. It is usually employed as an index of complexity of natural objects, which is suitable for a damage index in structural health monitoring [10]. The probabilistic reconstruction algorithm is defined as:

(13) $$P(x,y)=\sum_{i=1}^{N}p_{i}(x,y)=\sum_{i=1}^{N}I_{i}(\alpha -R)/(\alpha -1)$$

where *P*(*x*, *y*) is the defect probability at location (*x*, *y*). *p_i_*(*x*, *y*) is the estimation in the *i*th actuator-sensor path. *N* is the total number of actuator-sensor paths on laminate. Damage-sensitive feature *I* is the damage index. *α* is the scaling parameter controlling the size of the effective elliptical distribution area. For the *i*th path, *R* is the ratio of the total distance from the point (*x*, *y*) to the actuator (*x_ai_*, *Y_ai_*) and to the sensor (*x_si_*, *y_si_*), as follows:

(14) $$R=\left(\sqrt{{\left(x-x_{ai}\right)}^{2}+{\left(y-y_{ai}\right)}^{2}}+\sqrt{{\left(x-x_{si}\right)}^{2}+{\left(y-y_{si}\right)}^{2}}\right)/\sqrt{{\left(x_{ai}-x_{si}\right)}^{2}+{\left(y_{ai}-y_{si}\right)}^{2}}$$

**Figure 10.**
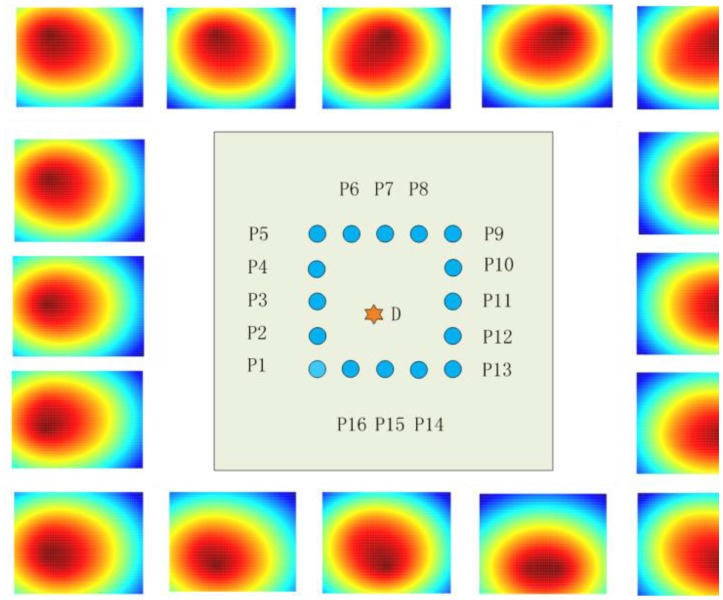
Sixteen tomography maps at each transducer array spot based on probabilistic reconstruction algorithm.

**Figure 11.**
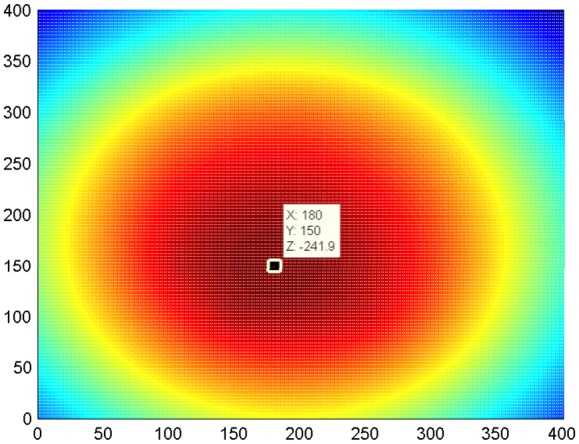
Thresholding fusion tomography map of denoising signal, based on probabilistic reconstruction algorithm; evaluated damage location: (180,150) (unit: mm).

**Figure 12.**
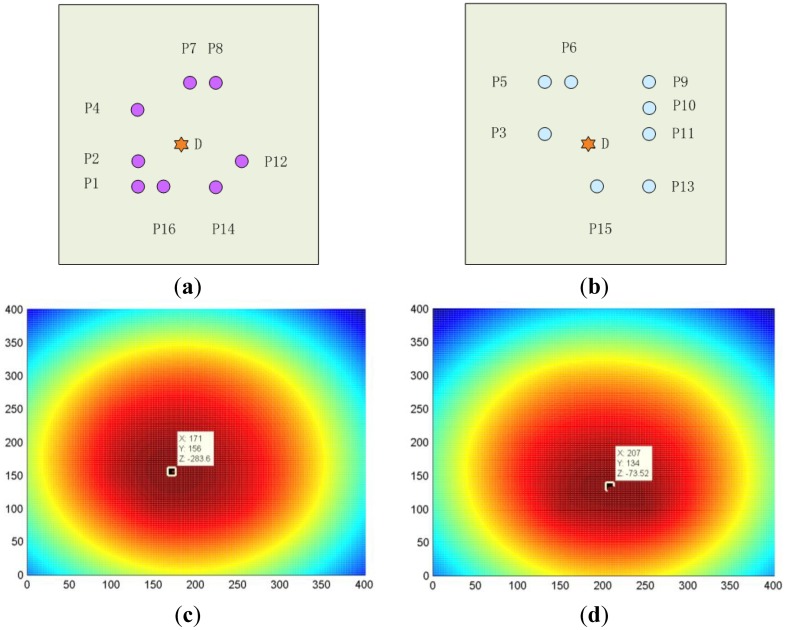
The tomography of the transducer array sparse optimizing results. (**a**) The top 8 transducers among the 16 all; (**b**) The relatively bad 8 transducers among the 16 all; (**c**) The top 8 transducers tomography evaluated damage location: (171,156); (**d**) The relatively bad 8 transducers tomography evaluated damage location: (207,134); (unit: mm).

As an example, after probabilistic reconstruction algorithm operations, the tomography maps of the raw signal as excitation at each transducer array spot of an experiment are shown as single maps' figures in Figure 10. The thresholding fusion map of their denoising signals is shown in Figure 11. The tomography of the transducer array sparse optimizing results is illustrated in Figure 12.

Clearly, the center of the damage was located as the top value point from the fusion tomography map. It proved the ability of the proposed approach to identify the correct position of the simulated damage (*i.e.*, mass of load) on a composite panel with anisotropic character.

#### 5.2.3. Experimental Error Analysis

According to the real damage location (175, 148), the damage location identification result error among three manners of transducers configuration were computed through Equations (15) and (16). The results are listed in Table 2.

(15) $$x_{ei}=[(x_{i}-x_{0})/x_{0}]\times 100\%$$

(16) $$y_{ei}=[(y_{i}-y_{0})/y_{0}]\times 100\%$$

Table 2 indicates that the proposed sensor array sparse optimization of composite laminates for wind turbine blades can relatively greatly reduce the number of transducer array spots. It guarantees more qualified monitoring for the relatively frequent occurrence damage area of the composite laminates for wind turbine blades. The statistics of 30 groups of experiments for this sensor array sparse optimization method also verified the same results and effectiveness of the method.

## 6. Summary and Conclusions

In this study, after illustrating the view of the wind energy industry development and its crucial requirement for SHM, a pre-processing IRSGWT algorithm based on neighboring coefficients is introduced for denoising composite laminate propagation signals. The method could effectively identify and extract weak signals sampled from composite structures, and improve the denoising performance indexes. It also overcomes the drawbacks of conventional wavelet methods that lose information in transforms and the shortcoming of IRSGWT denoising that causes error propagation.

What's more, sensor array sparse optimization of composite laminate for wind turbine blades is proposed that reduces the array transducer number from sixteen to eight, but the optimized eight transducers array layout owns greatly close accuracy as full sixteen transducers array and has higher accuracy than the not optimized eight transducers array. After the IRSGWT denoising for the lamb wave signals, the sparse optimized array with top eight transducers has good accuracy in tomography maps on locating the correct position of simulated damage (*i.e.*, mass of load) on composite laminates with anisotropy character than the not optimized array. The proposed method is verified in the experiment and it is helpful in the quantitative damage localization tomography performance of the composite laminates structure with multi-sensors.

In the following work, further study would focus on studying the pre-processing approaches for propagation signals in a practical wind turbine blade with composite laminates, and improving the accuracy of damage localization by investigate the mechanism of wave propagation in composite laminates blade with anisotropy character.

## Figures and Tables

**Table 1.** The denoising performance indexes of denoising results for the Lamb wave signal.

**Signals**		**SNR (dB)**	**MSE**	**CC**
Raw signal with noise		26.43592	0.07023	0.99619

SGWT	Hard	29.24623	0.04003	0.99734
	Soft	33.34943	0.03490	0.99914
	NeighCoef	35.73294	0.02949	0.99959

RSGWT	Hard	33.26719	0.03138	0.99898
	Soft	35.95234	0.03246	0.99920
	NeighCoef	38.98614	0.02819	0.99967

**IRSGWT**	Hard	36.23492	0.02723	0.99912
	Soft	40.34507	0.02804	0.99934
	**NeighCoef**	**42.14940**	**0.02239**	**0.99978**

**Table 2.** The damage location identification accuracy comparing among three manners of transducer configuration.

**Setup**	*x_error_*	*y_error_*
16 Transducers	2.9%	1.4%
Top 8 transducers optimized	2.3%	5.4%
Remaining 8 transducers optimized	17.3%	10.1%
